# Academic Achievement, Self-Concept, Personality and Emotional Intelligence in Primary Education. Analysis by Gender and Cultural Group

**DOI:** 10.3389/fpsyg.2019.03075

**Published:** 2020-01-22

**Authors:** Lucía Herrera, Mohamed Al-Lal, Laila Mohamed

**Affiliations:** ^1^Department of Developmental and Educational Psychology, University of Granada, Melilla, Spain; ^2^Early Childhood and Primary Education School “Pedro de Estopiñán”, Melilla, Spain

**Keywords:** academic achievement, self-concept, personality, emotional intelligence, gender, cultural group

## Abstract

A review of the scientific literature shows that many studies have analyzed the relationship between academic achievement and different psychological constructs, such as self-concept, personality, and emotional intelligence. The present work has two main objectives. First, to analyze the academic achievement, as well as the self-concept, personality and emotional intelligence, according to gender and cultural origin of the participants (European vs. Amazigh). Secondly, to identify what dimensions of self-concept, personality and emotional intelligence predict academic achievement. For this, a final sample consisting of 407 students enrolled in the last 2 years of Primary Education were utilized for the study. By gender, 192 were boys (47.2%) and 215 girls (52.8%), with an average age of 10.74 years old. By cultural group, 142 were of European origin (34.9%) and 265 of Amazigh origin (65.1%). The academic achievements were evaluated from the grades obtained in three school subjects: Natural Sciences, Spanish Language and Literature, and Mathematics, and the instruments used for data collection of the psychological constructs analyzed were the Self-Concept Test-Form 5, the Short-Form Big Five Questionnaire for Children, and the BarOn Emotional Quotient Inventory: Youth Version-Short. Based on the objectives set, first, the grades in the subject of Spanish Language and Literature varied depending on the gender of the students. Likewise, differences were found in self-concept, personality, and emotional intelligence according to gender. Also, the physical self-concept varied according to the cultural group. Regarding the second objective, in the predictive analysis for each of the subjects of the curriculum of Primary Education, the academic self-concept showed a greater predictive value. However, so did other dimensions of self-concept, personality and emotional intelligence. The need to carry out a comprehensive education in schools that addresses the promotion of not only academic but also personal and social competences is discussed. Also, that the study of the variables that affect gender differences must be deepened.

## Introduction

A review of the scientific literature has shown that many studies have analyzed the relationship between academic achievement and different psychological constructs such as self-concept (Susperreguy et al., 2018; Wolff et al., 2018; Sewasew and Schroeders, 2019), personality (Janošević and Petrović, 2019; Perret et al., 2019; Smith-Woolley et al., 2019), and emotional intelligence (Corcoran et al., 2018; Deighton et al., 2019; Piqueras et al., 2019). In this work, these psychological constructs are analyzed together with primary school children by gender and cultural group. Gender has been a highly studied variable since there are differences between boys and girls in academic performance as well as in the psychological constructs mentioned above (Chrisler and McCreary, 2010; Voyer and Voyer, 2014; Carvalho, 2016; Herrera et al., 2017; Janošević and Petrović, 2019). There are also studies that analyze the possible differences that may exist in the school context between children from different cultures (Schmitt et al., 2007; Strayhorn, 2010; Cvencek et al., 2018; Min et al., 2018). In this sense, there is a disadvantage in the school context for children of minority culture. The present study has been developed in Melilla, a Spanish city located in North Africa, close to Morocco. In their schools, children of European culture and children of Amazigh culture (also known as Berber) have been together from early childhood education. In addition, the predictive value of each of the dimensions that integrate self-concept, personality and emotional intelligence regarding the grades in three subjects of the Primary Education curriculum are analyzed. The psychological constructs analyzed in the present study are described below.

### Self-Concept

Many research studies have highlighted that the psychological construction of a positive self-concept by the students, during their academic stage, leads to success in educational environments and social and emotional situations (Eccles, 2009; Harter, 2012; Nasir and Lin, 2012; Chen et al., 2013). Therefore, the positive self-concept acquired in the formative years could help in the development of the strategies and skills needed for confronting life challenges (Huang, 2011). It has also been found that self-concept is positively associated with different factors such as the individual experiencing greater happiness (Hunagund and Hangal, 2014); a greater and better academic performance (Salami and Ogundokun, 2009); greater and more pro-social behaviors (Schwarzer and Fuchs, 2009); and lastly, an overall greater well-being (Mamata and Sharma, 2013).

Among the different models that link self-concept and academic performance, we found the Reciprocal Effects Model (REM), with a theoretical, methodological and empirical review conducted by Marsh and Martin (2011). This model argues that academic self-concept and performance mutually re-enforce themselves, with one producing advances in the other.

Starting with the evolution perspective, the Developmental Equilibrium Hypothesis has also been highlighted. The objective of this hypothesis is centered on achieving equilibrium between two factors that are directly related: self-concept and academic performance (Marsh et al., 2016a, b). Hence, achieving a state of equilibrium has important implications for the development of the individual, but it cannot be ignored that each individual’s development of self-concept is different depending on the personal, emotional, and social characteristics surrounding them (Eccles, 2009; Murayama et al., 2013; Paramanik et al., 2014).

The studies that relate self-concept with school or academic performance are exhaustive in the first educational stages as well as higher education (Guay et al., 2010; Möller et al., 2011; Skaalvik and Skjaalvik, 2013). The student’s self-concept, and the academic self-concept within it, has a strong influence on student self-efficacy (Ferla et al., 2009). Additionally, academic self-concept significantly correlates with school adjustment in Primary Education (Wosu, 2013; Mensah, 2014) and predicts academic achievement (Marsh and Martin, 2011; Guo et al., 2016). Therefore, in this research it is expected to find such predictive value.

The results from cross-cultural studies have shown that a negative self-concept had detrimental effects on the academic performance of the students from the different samples and countries (Marsh and Hau, 2003; Seaton et al., 2010; Nagengast and Marsh, 2012). Cvencek et al. (2018), when analyzing primary school students from a minority group and a majority group in North America, found that the academic performance, as well as the academic self-concept of the children from the minority group, were lower as compared to those from majority group. Similar results that show the disadvantage of minority groups in schools are found in other studies (Strayhorn, 2010). According to these results, it would be expected that in the present study children of Amazigh cultural origin obtained lower scores than those of European cultural origin in their academic performance and academic self-concept.

Another variable that has been analyzed along with self-concept and academic performance has been gender (Chrisler and McCreary, 2010; DiPrete and Jennings, 2012). Thus, in the meta-analysis study by Voyer and Voyer (2014), it was shown that a certain advantage in school performance existed in women, with their results showing differences in favor of the women for the Language subject. Differences according to gender were also found in self-concept (Nagy et al., 2010). Huang (2013), in a meta-analysis study, identified that the women had a greater self-concept in the subject matter or courses related to language, as well as the arts as compared to the men. Therefore, in this study we expect to find that girls obtain higher grades than boys in Spanish Language and Literature as well as academic self-concept.

### Personality

In general terms, personality and self-concept predict satisfaction with life (Parker et al., 2008). Also, personality moderates the effects of the frame of reference that are central for the shaping of self-concept (Jonkmann et al., 2012).

Within the models of personality, the Five Factor Model (McCrae and Costa, 1997) has been the most developed (Herrera et al., 2018), and it represents the dominant conceptualization of the structure of personality in current literature. It postulates that the five great factors of personality (emotional instability, extraversion, intellect/imagination, agreeableness, and conscientiousness) are found at the highest level in the hierarchy of personality.

Among the strongest arguments utilized to show that the measurements of personality, based on the Big-Five Factor Structure (Goldberg, 1990, 1992), correlate with academic performance, we find the evidence that supports the importance of the personality factors to predict behaviors that are socially valued and the recognition of personality as a component of the individual’s will (Chamorro-Premuzic et al., 2006). In this respect, the scientific literature shows studies that relate personality, through the five-factor model, with academic performance. Thus, agreeableness, and intellect/imagination (also known as openness) are related to academic performance (Poropat, 2009; Smith-Woolley et al., 2019). Specifically, conscientiousness predicts academic achievement (O′Connor and Paunonen, 2007), which is expected to be found in the present study.

Personality has been analyzed in different cultures (Allik et al., 2012). A good example of a broad study, which included 56 countries, is the one conducted by Schmitt et al. (2007). Among the main results, it was found that the five-factor structure of personality was robust among the main regions of the world. Also, the inhabitants from South America and East Asia were significantly different in their intellect/imagination characteristics as compared to the rest of the world regions. Thus, while the South American and European countries tended to occupy a higher position in openness, the cultures from East Asia were found in lower positions. This is attributed, among other factors, in that the Asian cultures are more collective, so that the openness dimension could be difficult to clearly identify, as proposed in the starting theoretical model. Based on these results, differences in personality dimensions are expected to be found among children of European and Amazigh cultural origin.

As for gender, differences have also been found. For example, the academic achievement in Primary Education is related to a higher conscientiousness in girls than in boys (Janošević and Petrović, 2019).

### Emotional Intelligence

Another factor that should be taken into account, as related to the academic achievements and school adjustment, is the emotional intelligence (EI). The models or theoretical approaches of EI are different (Cherniss, 2010; Herrera et al., 2017). On the one hand, models have been identified that are based on the processing of emotional information, which are focused on basic emotional abilities (Brackett et al., 2011). On the other hand, mixed models of EI have also been identified, which involve both intellectual and personality factors. The socio-emotional competence model by Bar-On (2006) forms part of the second group. In it, different dimensions are identified: intrapersonal, interpersonal, stress management, adaptability, and general mood.

Numerous research studies have examined the relationship between EI and academic performance (Pulido and Herrera, 2017). They have also analyzed their relationship with other variables such as adjustment and permanence in the school context (Hogan et al., 2010; Szczygieł and Mikolajczak, 2017), coping styles (MacCann et al., 2011), the degree of social competence (Franco et al., 2017), and school motivation (Usán and Salavera, 2018).

Emotional intelligence has also been analyzed in groups with different ethnic or cultural origins (Dewi et al., 2017; Min et al., 2018), and according to gender, differences were found in EI as well. Thus, for example, Herrera et al. (2017) obtained results that showed that girls in primary schools in Colombia exceeded the boys in the interpersonal dimension, while the boys stood out in the adaptability dimension. Similarly, Ferrándiz et al. (2012) identified that Spanish girls had higher scores in the interpersonal dimensions and the boys had higher scores in adaptability and general mood. Accordingly, we expect to find differences in emotional intelligence based on the cultural origin and gender of primary school children in this study.

As a function of what has been described until now, the present work has two main objectives. Firstly, to analyze the academic performance, as well as self-concept, personality and emotional intelligence, as a function of gender and cultural origin (European vs. Amazigh) of the participants. It is important to mention that the research study took place in the autonomous city of Melilla, a Spanish city that neighbors Morocco, with unique social, cultural and economic characteristics. In it, people from different cultures co-habit: European, Amazigh (also known as Berber, and who come from the Moroccan Rif), Sephardic and Hindu, although the majority of the population is of European and Amazigh descent and culture. The children with an Amazigh culture origin cohabit live and grow between their maternal culture, which counts with the Tamazight (a dialect that is orally transmitted) as a means of communication, and the European culture, with Spanish being the language employed at school and administrative environments of the city (Herrera et al., 2011).

Secondly, to identify which dimensions of self-concept, personality and emotional intelligence predict academic performance.

In addition, different hypotheses are raised based on the results found in the scientific literature that addresses the research topics described above.

*Hypothesis 1*. Academic grades differ depending on the gender and cultural origin of students. Thus, for example, as indicated by Voyer and Voyer (2014), girls will achieve higher grades than boys in the subject of Spanish Language and Literature. Likewise, children of cultural origin different from the school (i.e., children of Amazigh culture) will obtain lower grades than Spanish children (Strayhorn, 2010).

*Hypothesis 2*. The psychology constructs evaluated (self-concept, personality and emotional intelligence) differ according to gender and cultural origin. Among other issues, it is expected to find that girls have a higher academic self-concept than boys (Chrisler and McCreary, 2010), higher scores in the personality dimension of conscientiousness (Janošević and Petrović, 2019) as well as in the interpersonal EI dimension (Ferrándiz et al., 2012; Herrera et al., 2017). Likewise, children of European cultural origin are expected to obtain higher scores than those of Amazigh cultural origin in academic self-concept (Cvencek et al., 2018), intellect/imagination (Schmitt et al., 2007) and in the intrapersonal and interpersonal EI dimensions (Dewi et al., 2017; Min et al., 2018).

*Hypothesis 3*. Academic self-concept (Marsh and Martin, 2011; Guo et al., 2016), conscientiousness (O′Connor and Paunonen, 2007) and adaptability (Hogan et al., 2010) predict academic achievement.

## Materials and Methods

### Participants

A non-probabilistic sampling was used. Initially, 422 Primary school students were included in the research study. Nevertheless, once the non-valid cases were eliminated, defined as those who did not complete the evaluation instruments, or whose scores did not comply to what was set, the final sample was comprised of 407 students. These students were enrolled in eight of the twelve public early childhood and primary education centers in the autonomous city of Melilla, Spain (see Table 1), with a minimum age of 10 and a maximum of 12 years old. The description of the participants according to cultural origin, gender, grade and age is presented in Table 2.

**TABLE 1:** Distribution of participants according to the center of early childhood and primary education.

**Centers**	***N***	**%**
Center 1	16	3.9
Center 2	19	4.7
Center 3	12	2.9
Center 4	117	28.7
Center 5	18	4.4
Center 6	41	10.1
Center 7	100	24.6
Center 8	84	20.6
Total	407	100.0

**TABLE 2:** Distribution of participants according to cultural origin, gender, grade, and age.

**Variables**	**Cultural origin**							
	**European**		**Amazigh**		**Total**		**Age**	
	***N***	**%**	***N***	**%**	***N***	**%**	***Mean***	***SD***
**Gender**								
Boy	69	35.9	123	64.1	192	47.2	10.77	0.70
Girl	73	34.0	142	66.0	215	52.8	10.70	0.63
**Grade**								
Fifth	59	30.4	135	69.6	194	47.7	10.23	0.47
Sixth	83	39.0	130	61.0	213	52.3	11.20	0.45
Total	142	34.9	265	65.1	407	100.0		
Age	*Mean* = 10.78		*Mean* = 10.71		*Mean* = 10.74			
	*SD* = 0.67		*SD* = 0.67		*SD* = 0.67			

The children of European cultural origin are mainly of Spanish nationality and Catholic religion. They were born in the autonomous city of Melilla or elsewhere in the Iberian Peninsula. Their parents were born in Melilla or have changed their residence to this city for professional reasons (mainly to work in public administration or in the army). Children of Amazigh cultural origin were born in the autonomous city of Melilla, so their nationality is Spanish, or they reside in that city. Many of them are Muslims and have family in Morocco so, given the short distance away, they usually travel at weekends or holidays to Moroccan cities close to Melilla. Rearing practices of children in families of each cultural group developed, among other things, based on cultural values and identities that define them. Thus, for example, the raising of children of Amazigh cultural origin is similar to that of children in the Rif region of Morocco. However, these same children socialize not only with children of their own cultural group but also with children of European cultural origin in a Spanish city, that is, the autonomous city of Melilla. The same can be indicated for children of European cultural origin.

### Instruments

#### Academic Achievement

The final grades of the students of the school subjects Natural Sciences, Spanish Language and Literature, and Mathematics were obtained through a registry, provided by the student’s teachers. These were classified as insufficient (0–4.9 points), sufficient (5–5.9 points), good (6–6.9 points), notable (7–8.9 points) and outstanding (9–10 points).

#### Self-Concept

A Self-Concept Test-Form 5 (AF-5, García and Musitu, 2001) was utilized. It is composed of 30 items that evaluate the self-concept of an individual in academic (e.g., “I do my homework well”), social (e.g., “I make friends easily”), emotional (e.g., “I am afraid of some things”), family (e.g., “I feel that my parents love me”) and physical (e.g., “I take good care of my physical health”) contexts. This form has to be answered according to an attributive scale ranging from 1 to 99, according to how the item adjusts to what the individual evaluated thinks of it. For example, if a phrase indicates “music helps human well-being” and the student strongly agrees, he/she would answer with a high number, such as 94. But if the student disagreed, he/she would choose a low number, for example 9. Esnaola et al. (2011), when analyzing the psychometric properties of this test in the Spanish population from 12 to 84 years old, indicated that its total reliability was α = 0.74. The index of internal consistency, *Cronbach’s alpha*, calculated for the present work, had a value of α = 0.795. Also, its factorial or construct validity was corroborated in other research works (Elosua and Muñiz, 2010; Malo et al., 2011).

#### Personality

For the evaluation of personality, the Short-Form Big Five Questionnaire for Children (S-BFQ-C, Beatton and Frijters, 2012) was utilized. It is based on the model of personality structured by five factors (Big-Five Factor Structure), formulated by Goldberg (1990, 1992). These factors are denominated as emotional instability (e.g., “I am often sad”), extraversion (e.g., “I make friends easily”), intellect/imagination (e.g., “When the teacher explains something, I understand immediately”), agreeableness (e.g., “I share my things with other people”) and conscientiousness (e.g., “During class I concentrate on the things I do”), creating the Big Five Questionnaire-Children (BFQ-C). This questionnaire, is directed at children aged between 9 to 15 years old, and was designed and validated by Barbaranelli et al. (2003). In its initial version, its psychometric properties were analyzed with Italian children, although there are studies that have analyzed them in other populations such as for example the German (Muris et al., 2005), Spanish (Carrasco et al., 2005) or Argentinian (Cupani and Ruarte, 2008) populations. Nevertheless, one of the problems of this instrument is its length, given that is composed by 65 items, 13 for each scale. This is the reason why Beatton and Frijters (2012), in a broader study that sought to measure the effects of personality and satisfaction with life on the happiness of Australian youth aged from 9 to 14 years old, reduced the BFQ-C to a shorter version. This shorter version, named S-BFQ-C, is composed by 30 items, so that each of the scales is composed by 6 items. In this version, the questions have to be answered using a *Likert*-type scale with 5 response options (1 = Almost never; 5 = Almost always). The reliability, measured with *Cronbach’s Alpha*, was found to be between 0.60 and 0.80 for each of the five scales. For the present study, the total reliability found was α = 0.783.

#### Emotional Intelligence

The BarOn Emotional Quotient Inventory: Youth Version-Short (EQ-i: YV-S, Bar-On and Parker, 2000) was used. It is directed at children aged from 7 to 18 years old, and is composed of 30 items which have to be answered with a *Likert* scale with four possible responses (1 = Very seldom or Not true of me, 4 = Very often or True of me). Six items shape each of the following scales: intrapersonal (e.g., “It is easy to tell people how I feel”), interpersonal (e.g., “I care what happens to other people”), adaptability (e.g., “I can come up with good answers to hard questions”), stress management (e.g., “I can stay calm when I am upset”), and positive impression (e.g., “I like everyone I meet”). This last scale is useful for eliminating the cases of high social desirability. The sum of the first four scales provides the total EQ.

The reliability or internal consistency of the EQ-i YV-S scale oscillates between 0.65 and 0.87 (Bar-On and Parker, 2000). For this study, the total reliability was α = 0.745. Its internal structure was confirmed in Spanish (Esnaola et al., 2016), Hungarian (Kun et al., 2012), Mexican (Esnaola et al., 2018b), English (Davis and Wigelsworth, 2018) and Chinese (Esnaola et al., 2018a) populations.

### Procedure

#### Information Collection

In the first place, the participation of the management teams of the 12 early childhood and primary school education centers in Melilla was solicited. Of these, eight centers answered affirmatively. Afterward, within each center, the professor-tutor from each class or classes interested were contacted. A group meeting was conducted with the parents from each group-class, where information was provided about the objectives of the research study. The authorization of the children’s parents for the exclusive use of the results obtained, for educational and scientific purposes, was requested.

Once this process was finished, a document was provided to the teachers-tutors of each participating class which explained how to access the web program utilized for the management of the student’s grades in order to download this information in pdf format. Once this information was downloaded, they were asked to write down, in a double-entry table provided for each student, the final grades obtained in the subjects of Natural Sciences, Spanish Language and Literature, and Mathematics, using the scoring system of insufficient, sufficient, good, notable or outstanding. Teachers provided students’ grades to researchers at the end of the academic year.

The AF-5, the S-BFQ-C and the EQ-i: YV-S questionnaires were administered in the first school term to the students in fifth and sixth grade of Primary Education, collectively according to group-class. The maximum time provided for this was 55 min. Previously, the students were told that there were no right or wrong answers, and that they should answer with total sincerity, given that the test was anonymous. Also, that they should not write their name; and that what they were about to answer did not have any relation with the school grades; and lastly, that they should read the questions, and if they had any doubts (for example, not understanding a term), they should raise their hand so that the question could be resolved.

In order to be able to relate the results of the evaluation of the different psychological constructs and the academic grades, the teacher of each class assigned a number to each student. This number was recorded both in the grades provided by him/her and on the first page of each of the questionnaires administered.

#### Statistical Analysis of the Data

Before proceeding with the statistical analysis, from the 422 students who participated, it was determined if there were students who had not completed the three evaluation tests, and also if they obtained high scores in the positive impression scale of the EQ-i: YV-S. This resulted in the elimination of 15 individuals, resulting in a final sample of 407 students.

The statistical program *IBM SPSS Statistics 23* was used to carry out the statistical analysis. Descriptive statistics were utilized to describe the data (frequencies, percentages, mean and standard deviation). In other words, to answer the first research objective and the first two hypotheses, two Analysis of variance (ANOVA) were performed in which the Academic achievement was used as the dependent variable in one case, and self-concept, personality and EI as dependent variables in the other. In both cases, the independent variables were gender (boy or girl) and cultural group (European vs. Amazigh). The effect size was calculated with the partial eta-squared as the *post hoc* test, through the use of the *Bonferroni* test.

To address the second objective and the third hypothesis, three multiple linear regression analysis (with the enter method) were conducted, in which each subject was introduced as the dependent variable, with the predictive variables being the different dimensions which comprised the self-concept, personality and EI constructs. To justify the method used, the non-autocorrelation of the data was determined, using the Durbin Watson test, and the non-existence of multicollinearity, through the Variance Inflation Factor.

## Results

### Academic Achievement by Gender and Cultural Group

All the subjects had a maximum of five points, and were scored as: 1 = Insufficient, 2 = Sufficient, 3 = Good, 4 = Notable, 5 = Outstanding. The mean grade in Natural Sciences was 3.26 (*SD* = 1.33), for Spanish Language and Literature it was 3.33 (*SD* = 1.24) and in Mathematics, it was 3.19 (*SD* = 1.25).

Academic achievement as a function of the student’s gender and cultural group is presented in Table 3. The analysis of variance performed as a function of gender and cultural group showed that there were differences according to gender for the subject Spanish Language and Literature, *F* = 5.812, *p* = 0.016, *Eta2p* = 0.014, so that the girls obtained higher grades than the boys, *t* = 0.313, *p* = 0.016. No differences were found neither in Nature Sciences, *F* = 0.763, *p* = 0.383, *Eta2p* = 0.002, nor Mathematics, *F* = 1.692, *p* = 0.194, *Eta2p* = 0.004. On their part, no differences were found as a function of the cultural group, *F*_Natural Sciences_ = 0.376, *p* = 0.540, *Eta2p* = 0.001; *F*_Language and Literature_ = 0.565, *p* = 0.453, *Eta2p* = 0.001; *F*_Mathematics_ = 0.576, *p* = 0.448, *Eta2p* = 0.001.

**TABLE 3:** Academic achievement by gender and cultural group.

**Subjects**	**Gender**	**Cultural group**	***Mean***	***SD***	***N***
Natural sciences	Boy	Amazigh	3.10	1.41	123
		European	3.31	1.11	69
		Total	3.17	1.31	192
	Girl	Amazigh	3.35	1.26	142
		European	3.31	1.50	73
		Total	3.33	1.34	215
	Total	Amazigh	3.23	1.33	265
		European	3.31	1.32	142
		Total	3.26	1.33	407
Spanish language	Boy	Amazigh	3.13	1.29	123
and literature		European	3.24	1.24	69
		Total	3.17	1.27	192
	Girl	Amazigh	3.45	1.16	142
		European	3.54	1.27	73
		Total	3.48	1.20	215
	Total	Amazigh	3.30	1.23	265
		European	3.39	1.26	142
		Total	3.33	1.24	407
Mathematics	Boy	Amazigh	3.09	1.27	123
		European	3.15	1.20	69
		Total	3.11	1.24	192
	Girl	Amazigh	3.22	1.22	142
		European	3.36	1.33	73
		Total	3.27	1.25	215
	Total	Amazigh	3.16	1.24	265
		European	3.26	1.27	142
		Total	3.19	1.25	407

### Self-Concept, Personality and EI by Gender and Cultural Group

The analysis of variance results (see Supplementary Table S1) showed that there were significant differences as a function of gender for self-concept, more specifically in academic self-concept, with the girls achieving higher grades in *post hoc* comparisons using the *Bonferroni* test, *t* = 0.667, *p* = 0.007, and self-esteem, *t* = 1.139, *p* < 0.001, where the boys stood out. Likewise, differences were found in personality in favor of the girls within the conscientiousness, *t* = 1.136, *p* = 0.018, and agreeableness dimensions, *t* = 1.641, *p* = 0.001. Also, with respect to the EI, the girls had a higher score in the interpersonal scale, *t* = 1.016, *p* = 0.007, while the boys had a higher score in the stress management, *t* = 1.513, *p* < 0.001, and adaptability, *t* = 1.110, *p* = 0.008. Lastly, with respect to the analysis according to cultural group, there were only significant differences in the physical self-concept, with higher scores reached by the children of Amazigh cultural origin, *t* = 0.420, *p* = 0.036.

### Predictive Value of the Different Dimensions Evaluated With Respect to Academic Achievement

In first place, a linear regression analysis was conducted, where the dependent variable was the subject Natural Sciences and the predictive variables were the five dimensions of the self-concept, the five dimensions from personality and the four dimensions from EI (see Table 4). The model was significant with values *F* = 11.003, *p* < 0.001. Likewise, the coefficient of determination was *R*^2^ = 0.311 (adjusted *R*^2^ = 0.282). Durbin–Watson’s *d* test showed that there was no auto-correlation in the data (*d* = 1.583). Values of the Durbin Watson test between 1.5 and 2.5 indicate that the data are not correlated (Durbin and Watson, 1951). Also, the Variance Inflation Factor (VIF) obtained values lower than 5, so multicollinearity was not present (Berry and Feldman, 1985; Belsley, 1991).

**TABLE 4:** Regression analysis of the different dimensions analyzed with respect to the natural sciences subject.

**Dimensions**	**Non-standardized coefficients**		**Standardized coefficients**	***t***	***p***	**Collinearity tests**	
	***B***	***Standard error***	***Beta***			**TI**	**VIF**
Academic self-concept	0.297	0.037	0.490	7.940***	0.001	0.530	1.888
Social self-concept	0.018	0.052	0.020	0.344	0.731	0.592	1.689
Self-esteem	0.032	0.030	0.054	1.053	0.293	0.771	1.297
Family self-concept	0.110	0.055	0.108	1.993*	0.047	0.681	1.469
Physical self-concept	−0.235	0.040	–0.318	−5.810***	0.001	0.672	1.488
Conscientiousness	6.452E−5	0.020	0.000	0.003	0.997	0.505	1.979
Agreeableness	−0.028	0.019	–0.092	–1.450	0.148	0.496	2.017
Emotional instability	−0.004	0.016	–0.014	–0.252	0.801	0.691	1.447
Intellect/Imagination	0.037	0.018	0.120	2.025*	0.044	0.577	1.733
Extraversion	0.003	0.020	0.010	0.169	0.866	0.592	1.690
Intrapersonal	0.040	0.019	0.107	2.165*	0.031	0.822	1.217
Interpersonal	0.003	0.023	0.007	0.120	0.904	0.590	1.695
Stress management	0.010	0.020	0.029	0.497	0.619	0.580	1.723
Adaptability	0.029	0.021	0.082	1.377	0.170	0.562	1.781

***p < 0.001, *p < 0.05. TI, tolerance index; VIF, variance inflation factor.

In the order from greater to lesser predictive value, the dimensions were: academic self-concept, physical self-concept, intrapersonal, intellect/imagination, and family self-concept. The physical self-concept, as well as intrapersonal intelligence, negatively predicted the grades in Natural Sciences.

In second place, as related to the subject Spanish Language and Literature (see Table 5), the model was significant with values of *F* = 10.442, *p* < 0.001 and with a coefficient of determination of *R*^2^ = 0.299, adjusted *R*^2^ = 0.271. The data was not correlated (*d* = 1.672) and no multicollinearity was found.

**TABLE 5:** Regression analysis of the different dimensions analyzed with respect to the Spanish language and literature subject.

**Dimensions**	**Non-standardized coefficients**		**Standardized coefficients**	***t***	***p***	**Collinearity tests**	
	***B***	***Standard error***	***Beta***			**TI**	**VIF**
Academic self-concept	0.266	0.035	0.470	7.556***	0.001	0.530	1.888
Social self-concept	0.024	0.049	0.029	0.496	0.621	0.592	1.689
Self-esteem	0.014	0.028	0.025	0.481	0.631	0.771	1.297
Family self-concept	0.089	0.052	0.094	1.711	0.088	0.681	1.469
Physical self-concept	–0.170	0.038	–0.247	−4.471***	0.001	0.672	1.488
Conscientiousness	0.009	0.018	0.031	0.480	0.632	0.505	1.979
Agreeableness	–0.021	0.018	–0.075	–1.164	0.245	0.496	2.017
Emotional instability	–0.006	0.015	–0.020	–0.370	0.711	0.691	1.447
Intellect/Imagination	0.036	0.017	0.123	2.060*	0.040	0.577	1.733
Extraversion	0.021	0.018	0.067	1.144	0.253	0.592	1.690
Intrapersonal	0.037	0.017	0.107	2.138*	0.033	0.822	1.217
Interpersonal	0.000	0.022	0.001	0.016	0.987	0.590	1.695
Stress management	0.000	0.019	0.001	0.010	0.992	0.580	1.723
Adaptability	0.009	0.020	0.026	0.435	0.664	0.562	1.781

***p < 0.001, *p < 0.05. TI, tolerance index; VIF, variance inflation factor.

Once again, the academic self-concept dimension had the greatest predictive value, followed by the physical self-concept, intrapersonal intelligence, and intellect/imagination dimensions. The negative predictions remained the same.

In third and last place, for the subject of Mathematics (see Table 6), the model had a statistical significance, as shown by *F* = 10.790, *p* < 0.001. The coefficient of determination obtained was *R*^2^ = 0.306, adjusted *R*^2^ = 0.278. The data was not correlated (*d* = 1.600) and multicollinearity was not present.

**TABLE 6:** Regression analysis of the different dimensions analyzed with respect to the mathematics subject.

**Dimensions**	**Non-standardized coefficients**		**Standardized coefficients**	***t***	***p***	**Collinearity tests**	
	***B***	***Standard error***	***Beta***			**TI**	**VIF**
Academic self-concept	0.273	0.035	0.480	7.759***	0.001	0.530	1.888
Social self-concept	–0.004	0.049	–0.005	–0.078	0.938	0.592	1.689
Self-esteem	–0.021	0.028	–0.038	–0.744	0.458	0.771	1.297
Family self-concept	0.069	0.052	0.073	1.330	0.185	0.681	1.469
Physical self-concept	–0.189	0.038	–0.273	−4.968***	0.001	0.672	1.488
Conscientiousness	0.039	0.018	0.133	2.105*	0.036	0.505	1.979
Agreeableness	0.013	0.018	0.046	0.714	0.476	0.496	2.017
Emotional instability	–0.018	0.015	–0.067	–1.240	0.216	0.691	1.447
Intellect/Imagination	0.045	0.017	0.153	2.575*	0.010	0.577	1.733
Extraversion	0.012	0.018	0.038	0.647	0.518	0.592	1.690
Intrapersonal	0.009	0.017	0.026	0.529	0.597	0.822	1.217
Interpersonal	0.018	0.022	0.049	0.839	0.402	0.590	1.695
Stress management	0.002	0.019	0.006	0.104	0.917	0.580	1.723
Adaptability	0.054	0.020	0.162	2.693**	0.007	0.562	1.781

***p < 0.001, **p < 0.01, *p < 0.05. TI, tolerance index; VIF, variance inflation factor.

The predictive dimensions were academic self-concept, physical self-concept (in a negative manner), adaptability, intellect/imagination, and conscientiousness.

## Discussion

Based on the hypotheses set, first, the grades of the Spanish Language and Literature school subject varied depending on the gender of the students, which coincided with the results from other studies, which highlighted the girls’ higher grades (Huang, 2013; Voyer and Voyer, 2014). In this regard, it could be argued that academic and social expectations are different depending on gender (Voyer and Voyer, 2014). Likewise, the influence of socialization on the formation of gender behaviors must be taken into account in accordance with the cultural norms of masculinity and femininity (Gibb et al., 2008). Gender differences in academic achievement remain between different countries, regardless of their political, economic or social equality (Stoet and Geary, 2015). However, it is noteworthy that in adulthood women occupy fewer representations of political, economic and academic leadership than men.

Contrary to expectations (Strayhorn, 2010; Whaley and Noël, 2012), children of Amazigh origin did not obtain lower grades than those of European origin. These results may be due to the fact that in the city of Melilla children of both cultures are educated from early childhood education in schools where the language used is Spanish. Thus, the academic performance at the end of Primary Education does not differ depending on the cultural origin of the students. However, it is necessary to show that early childhood teachers dedicate great efforts so that children of Amazigh cultural origin develop the linguistic skills necessary for the correct learning and use of the Spanish language (Herrera et al., 2011). Therefore, hypothesis 1 is partially confirmed. That is, the results found indicate that academic achievement varies according to gender but not the cultural origin of the students.

Likewise, differences were found according to gender in self-concept, specifically in the academic self-concept and self-esteem; for personality, within the factors of conscientiousness and agreeableness; in addition to emotional intelligence, particularly in the interpersonal, stress management and adaptability scales. As for the differences found for self-concept according to gender (Nagy et al., 2010), the results found for academic self-concept showed differences in favor of the girls (Malo et al., 2011). Nevertheless, other factors should be taken into account, such as the academic responsibilities associated to school success and failure, given that, for example, the boys in Compulsory Secondary Education attribute their academic success to their skills, while the girls attribute them to their effort (Inglés et al., 2012). As for emotional self-concept or self-esteem, the boys exceeded the girls (Xie et al., 2019). Cross-cultural studies show that differences in self-esteem according to gender are maintained in different countries, although their magnitude differ according to the cultural differences found in the socioeconomic, sociodemographic, gender equality and cultural value indicators (Bleidorn et al., 2016). In this respect, the emotion literacy programs, based on the development of emotional intelligence, could be a useful tool for the development of self-esteem (Cheung et al., 2014).

As for the differences in the personality dimensions conscientiousness and agreeableness in favor of the girls, the results were in agreement with previous studies (Rahafar et al., 2017; Janošević and Petrović, 2019). Within the differences in EI according to gender, the girls scored higher in the interpersonal scale, while the boys did so in stress management and adaptability (Ferrándiz et al., 2012; Herrera et al., 2017). In this way, the girls showed competencies and skills that were higher than the boys in empathy, social responsibility, and interpersonal relationships. On the contrary, the boys stood out in stress tolerance and impulse control (stress management), as well as in reality-testing, flexibility, and problem-solving (adaptability). These differences, as a function of gender, could be due to cultural factors and family rearing practices differentiated as a function of gender (Joseph and Newman, 2010).

Also, the physical self-concept varied according to the cultural origin, where children from the Amazigh culture obtained higher scores than children of European culture origin. This may be due to the influence of cultural values (their own, meaning Amazigh, as well as the context in which they live in, given that the children are socialized in a European context), with respect to body image and physical self-concept (Marsh et al., 2007).

Based on the results found, the second hypothesis is partially confirmed. The three psychological constructs evaluated differ according to gender in the expected direction but only in the self-concept are differences found according to the cultural origin. Although it was expected to find differences in favor of children of European cultural origin in academic self-concept (Cvencek et al., 2018), they have been found in physical self-concept in favor of children of Amazigh cultural origin. As previously indicated, children of European and Amazigh culture develop in the same school contexts from the early educational stages. Thus, educational policies developed in schools may have contributed to eliminating the possible socio-cultural disadvantages of children of Amazigh cultural origin. This implies, therefore, that there are no differences depending on the cultural group in the academic self-concept.

In the predictive analysis developed for each of the school subjects of the curriculum of Primary Education, with the aim of answering the second objective and the third hypothesis of the study, the academic self-concept showed a greater predictive value (Marsh and Martin, 2011; Jansen et al., 2015; Guo et al., 2016; Lösch et al., 2017; Susperreguy et al., 2018). This result confirms the third hypothesis. That is, the relevance of academic self-concept in school performance. However, so did other dimensions of self-concept. More specifically, the physical self-concept negatively predicted the academic results in the three subjects evaluated (Lohbeck et al., 2016). Children who participated in the study are in the process of transition from childhood to adolescence. Biological changes in their bodies due to this stage of evolutionary development as well as greater attention to appearance and physical abilities may interfere at the end of Primary Education in their academic performance. Furthermore, the family self-concept predicted the grades of the Natural Sciences school subject. This last result points to the influence of the family on self-concept as well as academic results (Corrás et al., 2017; Mortimer et al., 2017; Häfner et al., 2018).

Personality also predicted the academic results in the three school subjects from the Primary Education curriculum analyzed (O′Connor and Paunonen, 2007; Spengler et al., 2016; Bergold and Steinmayr, 2018), i.e., the intellect/imagination dimension for the three subjects and conscientiousness for Mathematics. In the first case, it may be because intellect/imagination or openness is a personality dimension that reflects cognitive exploration (DeYoung, 2015). It refers to the ability and tendency to find, understand and use complex patterns of both sensory and abstract information. Therefore, those children who score higher in intellect/imagination will get better academic results than those with lower scores. In the second case, conscientiousness relates to responsibility, persistence, trustworthiness, and being purposeful (Conrad and Patry, 2012). Children with high conscientiousness can develop a variety of effective learning strategies, which may be associated with higher academic performance in Mathematics.

Likewise, EI predicted academic achievement in every case (Salami and Ogundokun, 2009; Hogan et al., 2010; Brackett et al., 2011; MacCann et al., 2011). More specifically, the intrapersonal scale predicted it for the subjects of Natural Sciences and Spanish Language and Literature. Intrapersonal intelligence involves the knowledge and labeling of one’s own feelings. This ability may contribute to achieving better grades in both subjects of the curriculum. For example, in the subject of Spanish Language and Literature it can facilitate the communicative skills related to the reading of different kinds of texts, their reflection and their understanding. On the other hand, in the subject of Nature Sciences it can contribute to interpret reality in order to address the solution to the different problems that arise, as well as to explain and predict natural phenomena and to face the need to develop critical attitudes before the consequences that result from scientific advances. In the case of the Mathematics subject, the adaptability scale predicted the academic achievement. Adaptability implies abilities such as being able to adjust one’s emotions and behaviors to changing situations or conditions, which is closely related to mathematical thinking.

In general, scientific literature shows that academic achievement is related to self-concept (Susperreguy et al., 2018; Wolff et al., 2018; Sewasew and Schroeders, 2019), personality (Perret et al., 2019; Smith-Woolley et al., 2019), and EI (Corcoran et al., 2018; Deighton et al., 2019; Piqueras et al., 2019). Also, that within these construct, academic self-concept (Ferla et al., 2009; Guay et al., 2010; Chen et al., 2013; Marsh et al., 2014), intellect/imagination (Poropat, 2009; Smith-Woolley et al., 2019), and adaptability (MacCann et al., 2011; Szczygieł and Mikolajczak, 2017) correlate significantly with academic achievement. In this research the predictive value of the dimensions of self-concept, personality and EI regarding the academic grades obtained in three subjects of the Primary Education curriculum has been established. One of its strengths is that it analyzes the predictive value of these psychological constructs together, not separately as in other studies.

In addition, the study has been developed in a multicultural context where children of European and Amazigh cultural origin coexist. Children of Amazigh cultural origin usually have access to early childhood education centers with a lower knowledge of the Spanish language than children of European cultural origin (Herrera et al., 2011). Although studies carried out with groups of cultural minorities show differences in their school performance (Strayhorn, 2010; Whaley and Noël, 2012), in the present study they are not at the end of Primary Education. This fact may be due to the linguistic policy developed in Melilla educational centers, which means that the mother language of children of Amazigh origin does not represent a disadvantage for academic achievement.

Further, gender differences found in the study seem to be more relevant than cultural differences. In fact, they are only in the physical self-concept in the latter case. Personality can mediate in adapting to school demands, so that girls are more conscientiousness than boys and follow norms in a more adaptive way (Carvalho, 2016). Moreover, since girls excel in their academic self-concept, their self-efficacy may also be superior to that of boys, which contributes to a better school adjustment (Ferla et al., 2009). Girls also have greater interpersonal intelligence, indicating better empathy, social responsibility and interpersonal relationships (Ferrándiz et al., 2012). Such non-cognitive abilities can stimulate the development of positive interpersonal relationships in the classroom with both the teachers and their peers. These individual differences may be due to family and social influences where, for example, girls are expected to be more emotionally expressive than boys (Meshkat and Nejati, 2017). In this same direction it could explain why children have greater self-esteem and stress management that girls.

### Practical Implications for Education

In light of the results obtained in the present research study, the need to carry out a comprehensive education in schools that addresses the promotion of not only academic but also personal, social and emotional competences, are underlined (Cherniss, 2010; Hunagund and Hangal, 2014; Herrera et al., 2017; Szczygieł and Mikolajczak, 2017; Corcoran et al., 2018; Cvencek et al., 2018). For this, the application of the principles derived from Positive Psychology in the education field would be an adequate strategy (Suldo et al., 2015; Chodkiewicz and Boyle, 2017; Domitrovich et al., 2017; Shoshani and Slone, 2017). Thus, intellectual, procedural and emotional aspects have to be worked on in learning, the latter being clear drivers of learning. The pleasant emotions experienced by children in educational settings will allow greater happiness and emotional well-being in them (Gil and Martínez, 2016). For it, teachers must be trained in good teaching practices that allow the interest of students to learn as well as guide them in the emotional domain (Castillo et al., 2013; Oberle et al., 2016; Conners-Burrow et al., 2017).

Likewise, schools must respond to the gender and cultural differences of students (Chrisler and McCreary, 2010; DiPrete and Jennings, 2012), particularly the first based on the results of this study. Thus, for example, the development of greater self-esteem in girls (Bleidorn et al., 2016; Xie et al., 2019) should be encouraged. As indicated by Cheung et al. (2014), emotional literacy programs that are based on emotional intelligence are an appropriate strategy for promoting self-esteem. Similarly, gender differences must be taken into account in response to other factors such as cultural group, family beliefs and parenting practices (Chrisler and McCreary, 2010; Joseph and Newman, 2010; Nagy et al., 2010; Allik et al., 2012; Marsh et al., 2015).

### Study Limitations and Proposal for Future Research

The present study has been developed taking into account only the last two school years of the education stage of Primary Education, just before the transition to Compulsory Secondary Education. Given that the scientific literature shows evolutionary changes in the development of the constructs analyzed (Huang, 2011; Murayama et al., 2013; Marsh et al., 2015; Bleidorn et al., 2016), longitudinal studies could be conducted in future research studies from Primary Education to Compulsory Secondary Education in order to determine the magnitude and direction of these changes.

On the other hand, all the instruments for data collection used to evaluate the psychological constructs analyzed in the present study are based on self-report measures. Different types of measuring instruments (self-report measures and performance measures) should be combined in future studies (Petrides et al., 2010; Mayer et al., 2012).

Gender differences in academic achievement as well as the psychological constructs analyzed have been revealed. However, it has to deepen the analysis of personal variables, family, social and cultural factors that contribute to that, even though women get better scores on their school performance across the different educational stages, at adulthood that reach fewer representations than men in leadership positions (Stoet and Geary, 2015).

Finally, given the cultural diversity in schools it is necessary to develop studies that analyze academic achievement as well as its relationship with different psychological variables in students of different cultural groups. Cross-cultural studies comparing different countries are necessary (Marsh and Hau, 2003; Nagengast and Marsh, 2012; Bleidorn et al., 2016; Min et al., 2018) but teachers have to know how to deal with coexistence and cultural diversity within the classrooms.

## Data Availability Statement

The datasets generated for this study are available on request to the corresponding author.

## Ethics Statement

The studies involving human participants were reviewed and approved by the Research Commission, Faculty of Educational Sciences and Sports, University of Granada, Melilla, Spain. Written informed consent to participate in this study was provided by the participants’ legal guardian/next of kin.

## Author Contributions

LH, MA-L, and LM shared conception, design, and the final version of the work, were jointly accountable for the content of the work, ensured that all aspects related to accuracy or integrity of the study were investigated and resolved in an appropriate way, and shared the internal consistency of the manuscript. MA-L and LM contributions were mainly in the theoretical part and in revising it critically. LH contribution was mainly in methodological question and data analysis.

## Conflict of Interest

The authors declare that the research was conducted in the absence of any commercial or financial relationships that could be construed as a potential conflict of interest.
