# Supplementary material for: Academic Achievement, Self-Concept, Personality and Emotional Intelligence in Primary Education. Analysis by Gender and Cultural Group
Source: Front Psychol. 2020 Jan 22;10:3075. doi: 10.3389/fpsyg.2019.03075 (PMC6987137; doi:10.3389/fpsyg.2019.03075)
Supplement: Supplementary file 1 [file Table_1.DOC]

Supplementary Material

# Academic Achievement, Self-Concept, Personality and Emotional Intelligence in Primary Education. Analysis by gender and cultural group

**Lucía Herrera^*^, Mohamed Al-Lal, Laila Mohamed**

*** Correspondence:** Lucia Herrera: luciaht@ugr.es

Table S1. Self-concept, personality and EI by gender and cultural group.

| Variables | Dimensions | Gender | Cultural group | *Mean* | *SD* | *F_gender_* | *p* | *Eta2p* | *F_cultural group_* | *p* | *Eta2p* |
| --- | --- | --- | --- | --- | --- | --- | --- | --- | --- | --- | --- |
| Self-concept | Academic self-concept | Boy | Amazigh | 7.27 | 2.15 | 7.471** | .007 | .021 | .023 | .879 | .000 |
|  |  |  | European | 7.12 | 2.48 |  |  |  |  |  |  |
|  |  |  | Total | 7.21 | 2.27 |  |  |  |  |  |  |
|  |  | Girl | Amazigh | 7.82 | 2.17 |  |  |  |  |  |  |
|  |  |  | European | 7.90 | 2.04 |  |  |  |  |  |  |
|  |  |  | Total | 7.85 | 2.12 |  |  |  |  |  |  |
|  |  | Total | Amazigh | 7.58 | 2.17 |  |  |  |  |  |  |
|  |  |  | European | 7.52 | 2.29 |  |  |  |  |  |  |
|  |  |  | Total | 7.56 | 2.21 |  |  |  |  |  |  |
|  | Social self-concept | Boy | Amazigh | 7.73 | 1.51 | .173 | .678 | .000 | 1.404 | .237 | .004 |
|  |  |  | European | 7.82 | 1.58 |  |  |  |  |  |  |
|  |  |  | Total | 7.76 | 1.53 |  |  |  |  |  |  |
|  |  | Girl | Amazigh | 7.69 | 1.53 |  |  |  |  |  |  |
|  |  |  | European | 8.00 | 1.42 |  |  |  |  |  |  |
|  |  |  | Total | 7.80 | 1.50 |  |  |  |  |  |  |
|  |  | Total | Amazigh | 7.71 | 1.52 |  |  |  |  |  |  |
|  |  |  | European | 7.91 | 1.49 |  |  |  |  |  |  |
|  |  |  | Total | 7.78 | 1.51 |  |  |  |  |  |  |
|  | Self-esteem | Boy | Amazigh | 6.31 | 2.05 | 21.154*** | <.001 | .057 | .000 | .997 | .000 |
|  |  |  | European | 6.58 | 2.14 |  |  |  |  |  |  |
|  |  |  | Total | 6.41 | 2.08 |  |  |  |  |  |  |
|  |  | Girl | Amazigh | 5.44 | 2.33 |  |  |  |  |  |  |
|  |  |  | European | 5.17 | 2.37 |  |  |  |  |  |  |
|  |  |  | Total | 5.35 | 2.34 |  |  |  |  |  |  |
|  |  | Total | Amazigh | 5.83 | 2.24 |  |  |  |  |  |  |
|  |  |  | European | 5.84 | 2.36 |  |  |  |  |  |  |
|  |  |  | Total | 5.83 | 2.28 |  |  |  |  |  |  |
|  | Family self-concept | Boy | Amazigh | 8.63 | 1.34 | 3.570 | .060 | .010 | .356 | .551 | .001 |
|  |  |  | European | 8.64 | 1.40 |  |  |  |  |  |  |
|  |  |  | Total | 8.63 | 1.36 |  |  |  |  |  |  |
|  |  | Girl | Amazigh | 8.83 | 1.31 |  |  |  |  |  |  |
|  |  |  | European | 8.99 | 1.18 |  |  |  |  |  |  |
|  |  |  | Total | 8.88 | 1.26 |  |  |  |  |  |  |
|  |  | Total | Amazigh | 8.74 | 1.32 |  |  |  |  |  |  |
|  |  |  | European | 8.82 | 1.30 |  |  |  |  |  |  |
|  |  |  | Total | 8.77 | 1.31 |  |  |  |  |  |  |
|  | Physical self-concept | Boy | Amazigh | 7.85 | 1.54 | 2.604 | .107 | .007 | 4.409* | .036 | .012 |
|  |  |  | European | 7.50 | 1.86 |  |  |  |  |  |  |
|  |  |  | Total | 7.72 | 1.67 |  |  |  |  |  |  |
|  |  | Girl | Amazigh | 7.60 | 1.84 |  |  |  |  |  |  |
|  |  |  | European | 7.10 | 2.03 |  |  |  |  |  |  |
|  |  |  | Total | 7.43 | 1.92 |  |  |  |  |  |  |
|  |  | Total | Amazigh | 7.71 | 1.71 |  |  |  |  |  |  |
|  |  |  | European | 7.29 | 1.95 |  |  |  |  |  |  |
|  |  |  | Total | 7.56 | 1.81 |  |  |  |  |  |  |
| Personality | Conscientiousness | Boy | Amazigh | 22.54 | 4.60 | 5.647* | .018 | .016 | .011 | .918 | .000 |
|  |  |  | European | 22.68 | 4.66 |  |  |  |  |  |  |
|  |  |  | Total | 22.60 | 4.61 |  |  |  |  |  |  |
|  |  | Girl | Amazigh | 23.87 | 4.05 |  |  |  |  |  |  |
|  |  |  | European | 23.63 | 3.97 |  |  |  |  |  |  |
|  |  |  | Total | 23.79 | 4.01 |  |  |  |  |  |  |
|  |  | Total | Amazigh | 23.28 | 4.34 |  |  |  |  |  |  |
|  |  |  | European | 23.18 | 4.32 |  |  |  |  |  |  |
|  |  |  | Total | 23.24 | 4.33 |  |  |  |  |  |  |
|  | Agreeableness | Boy | Amazigh | 19.97 | 4.64 | 11.356** | .001 | .031 | .011 | .916 | .000 |
|  |  |  | European | 19.80 | 5.31 |  |  |  |  |  |  |
|  |  |  | Total | 19.90 | 4.88 |  |  |  |  |  |  |
|  |  | Girl | Amazigh | 21.49 | 4.03 |  |  |  |  |  |  |
|  |  |  | European | 21.56 | 3.64 |  |  |  |  |  |  |
|  |  |  | Total | 21.51 | 3.89 |  |  |  |  |  |  |
|  |  | Total | Amazigh | 20.81 | 4.37 |  |  |  |  |  |  |
|  |  |  | European | 20.71 | 4.59 |  |  |  |  |  |  |
|  |  |  | Total | 20.78 | 4.44 |  |  |  |  |  |  |
|  | Emotional instability | Boy | Amazigh | 16.58 | 4.31 | .311 | .577 | .001 | 2.309 | .130 | .007 |
|  |  |  | European | 16.75 | 4.95 |  |  |  |  |  |  |
|  |  |  | Total | 16.65 | 4.54 |  |  |  |  |  |  |
|  |  | Girl | Amazigh | 15.70 | 4.55 |  |  |  |  |  |  |
|  |  |  | European | 17.07 | 4.62 |  |  |  |  |  |  |
|  |  |  | Total | 16.17 | 4.61 |  |  |  |  |  |  |
|  |  | Total | Amazigh | 16.09 | 4.46 |  |  |  |  |  |  |
|  |  |  | European | 16.92 | 4.76 |  |  |  |  |  |  |
|  |  |  | Total | 16.39 | 4.58 |  |  |  |  |  |  |
|  | Intellect/Imagination | Boy | Amazigh | 21.42 | 4.30 | 3.210 | .074 | .009 | .552 | .458 | .002 |
|  |  |  | European | 21.52 | 4.59 |  |  |  |  |  |  |
|  |  |  | Total | 21.46 | 4.40 |  |  |  |  |  |  |
|  |  | Girl | Amazigh | 20.32 | 4.25 |  |  |  |  |  |  |
|  |  |  | European | 20.92 | 3.95 |  |  |  |  |  |  |
|  |  |  | Total | 20.52 | 4.15 |  |  |  |  |  |  |
|  |  | Total | Amazigh | 20.81 | 4.30 |  |  |  |  |  |  |
|  |  |  | European | 21.21 | 4.26 |  |  |  |  |  |  |
|  |  |  | Total | 20.95 | 4.28 |  |  |  |  |  |  |
|  | Extraversion | Boy | Amazigh | 22.76 | 4.12 | .154 | .695 | .000 | .262 | .609 | .001 |
|  |  |  | European | 22.04 | 4.39 |  |  |  |  |  |  |
|  |  |  | Total | 22.49 | 4.22 |  |  |  |  |  |  |
|  |  | Girl | Amazigh | 22.10 | 3.64 |  |  |  |  |  |  |
|  |  |  | European | 22.36 | 4.08 |  |  |  |  |  |  |
|  |  |  | Total | 22.19 | 3.79 |  |  |  |  |  |  |
|  |  | Total | Amazigh | 22.39 | 3.87 |  |  |  |  |  |  |
|  |  |  | European | 22.21 | 4.22 |  |  |  |  |  |  |
|  |  |  | Total | 22.33 | 3.99 |  |  |  |  |  |  |
| Emotional Intelligence | Intrapersonal | Boy | Amazigh | 13.34 | 3.55 | .018 | .893 | .000 | .002 | .964 | .000 |
|  |  |  | European | 12.77 | 4.35 |  |  |  |  |  |  |
|  |  |  | Total | 13.12 | 3.86 |  |  |  |  |  |  |
|  |  | Girl | Amazigh | 12.84 | 3.39 |  |  |  |  |  |  |
|  |  |  | European | 13.37 | 3.22 |  |  |  |  |  |  |
|  |  |  | Total | 13.02 | 3.33 |  |  |  |  |  |  |
|  |  | Total | Amazigh | 13.06 | 3.46 |  |  |  |  |  |  |
|  |  |  | European | 13.08 | 3.80 |  |  |  |  |  |  |
|  |  |  | Total | 13.07 | 3.58 |  |  |  |  |  |  |
|  | Interpersonal | Boy | Amazigh | 17.36 | 2.96 | 7.414** | .007 | .021 | .059 | .808 | .000 |
|  |  |  | European | 17.68 | 3.86 |  |  |  |  |  |  |
|  |  |  | Total | 17.48 | 3.32 |  |  |  |  |  |  |
|  |  | Girl | Amazigh | 18.61 | 3.39 |  |  |  |  |  |  |
|  |  |  | European | 18.46 | 3.39 |  |  |  |  |  |  |
|  |  |  | Total | 18.56 | 3.38 |  |  |  |  |  |  |
|  |  | Total | Amazigh | 18.05 | 3.26 |  |  |  |  |  |  |
|  |  |  | European | 18.09 | 3.63 |  |  |  |  |  |  |
|  |  |  | Total | 18.07 | 3.39 |  |  |  |  |  |  |
|  | Stress management | Boy | Amazigh | 13.20 | 4.00 | 12.916*** | <.001 | .035 | 3.814 | .052 | .011 |
|  |  |  | European | 13.50 | 4.52 |  |  |  |  |  |  |
|  |  |  | Total | 13.31 | 4.19 |  |  |  |  |  |  |
|  |  | Girl | Amazigh | 11.17 | 3.32 |  |  |  |  |  |  |
|  |  |  | European | 12.51 | 3.56 |  |  |  |  |  |  |
|  |  |  | Total | 11.63 | 3.45 |  |  |  |  |  |  |
|  |  | Total | Amazigh | 12.07 | 3.77 |  |  |  |  |  |  |
|  |  |  | European | 12.99 | 4.06 |  |  |  |  |  |  |
|  |  |  | Total | 12.40 | 3.90 |  |  |  |  |  |  |
|  | Adaptability | Boy | Amazigh | 17.53 | 3.57 | 7.218** | .008 | .020 | .029 | .865 | .000 |
|  |  |  | European | 17.37 | 4.29 |  |  |  |  |  |  |
|  |  |  | Total | 17.47 | 3.84 |  |  |  |  |  |  |
|  |  | Girl | Amazigh | 16.19 | 3.59 |  |  |  |  |  |  |
|  |  |  | European | 16.50 | 3.63 |  |  |  |  |  |  |
|  |  |  | Total | 16.30 | 3.60 |  |  |  |  |  |  |
|  |  | Total | Amazigh | 16.79 | 3.63 |  |  |  |  |  |  |
|  |  |  | European | 16.92 | 3.97 |  |  |  |  |  |  |
|  |  |  | Total | 16.83 | 3.75 |  |  |  |  |  |  |

****p* < .001, ** *p* < .01, * *p* < .05
